# Calculation of pediatric femoral fracture rotation from direct roentgenograms

**DOI:** 10.1007/s10195-013-0244-0

**Published:** 2013-05-07

**Authors:** M. S. Ozel, I. E. Ketenci, E. Kaya, S. Tuna, B. Saygi

**Affiliations:** 1 Department of Orthopaedics and Traumatology, Haydarpasa Numune Education and Research Hospital, Tibbiye Cad. No:40, Uskudar, Istanbul, 34671 Turkey; 2 Department of Orthopaedics and Traumatology, Fatih Sultan Mehmet Hospital, Istanbul, Turkey; 3 Department of Orthopaedics and Traumatology, Umraniye Education and Research Hospital, Istanbul, Turkey

**Keywords:** Pediatric femoral fracture, Fracture rotation, Direct roentgenogram, Mathematical method

## Abstract

**Background:**

Radiologic determination of pediatric femoral fracture rotation has been debated. Measuring the antetorsion angle of the fractured femur by computed tomography and comparing it with the opposite side has been the method of choice for this purpose. However, no simple method for direct measurement of femoral fracture rotation exists in the literature. In this study, our aim was to test a mathematical method of measuring the axial plane malrotation from direct roentgenograms.

**Materials and methods:**

A pediatric femoral shaft fracture model was produced. The bone was secured to a wooden frame that allowed the distal part of the fracture to rotate around an axis. Radiographs were taken at known intervals of rotation ranging from the neutral position to 60° external rotation and to 60° internal rotation in 5° increments of rotation. Five independent, blinded observers measured the radiographs and calculated the fracture rotation according to a standard formula. Calculated rotation values were compared with known rotation values.

**Results:**

Calculated rotation values were close to actual rotation values throughout the arc of rotation. The mean absolute error of five observers for all measurements of external and internal rotation was 3.97° (±0.83). The correlation coefficient between calculated and actual rotation values was 0.9927. The interobserver intraclass correlation coefficient for calculated rotation was 0.997.

**Conclusions:**

Absolute error and correlation coefficient values indicate that this method is accurate and reliable in determining the fracture rotation.

## Introduction

Femoral shaft fractures are one of the most common lower extremity fractures in children [1]. Treatment options for these fractures range from closed reduction and hip spica casting with or without traction to surgical stabilization with intramedullary devices, plates and screws, and external fixators [2–4]. Management depends on various factors such as age, type of fracture, existence of additional trauma and the preference of the clinician [5]. Spica casting has been shown to be effective for most children younger than 6 years of age and it can be performed at an early stage or after a period of traction [2–4].

Several direct roentgenograms have to be taken during the traction period or during the follow-up with spica casts to assess the maintenance of the reduction. Angular deformities on coronal and sagittal planes can be determined easily with conventional roentgenograms but rotational deformities on the transverse plane cannot [6]. The amount of fracture rotation is determined indirectly by computed tomography, measuring the antetorsion angle and comparing it with the opposite side [7, 8]. Methods of measuring the antetorsion angle from direct roentgenograms have been reported [9, 10], but their accuracy has been disputed, and Norbeck et al. [11] suggested the use of computed tomographic scanning for greater accuracy.

Although it has been reported that up to 25°–30° of rotational malunion seems to be well tolerated [12], in most of the series that evaluated conservative management of pediatric femoral shaft fractures, rotational deformity exceeding 10° was considered to be unacceptable [6, 13]. Staheli and Sheridan [14] reported rotational malalignment in five of 20 patients and found that malalignment greater than 10° was symptomatic. In the study by Verbeek, it was reported that one-third of the children with femoral fractures, treated with conservative therapy, had significant rotational deformities between 10° and 30° [15]. So malrotation is a problem in the management of pediatric femoral shaft fractures. Despite this, in some studies, malrotation is only evaluated clinically and rotation angles are not given [16]. This shows that there is a need for an easier method for assessment of malrotation, which could be performed at each visit during the follow-up. A direct roentgenogram would be an ideal imaging study for this purpose because it is available everywhere, is easy to perform, causes less radiation exposure than CT and is less expensive.

Calculation of rotational deformity on a direct roentgenogram was performed by Henderson et al. [17] on a pediatric supracondylar fracture model. However, no simple method for the assessment of fracture rotation of long tubular bones exists in the literature. In this study, our aim was to test a mathematical method of measuring axial plane malrotation in pediatric femoral shaft fractures, which could also be applied to any long tubular bone fracture.

## Materials and methods

**Fig. 1:**
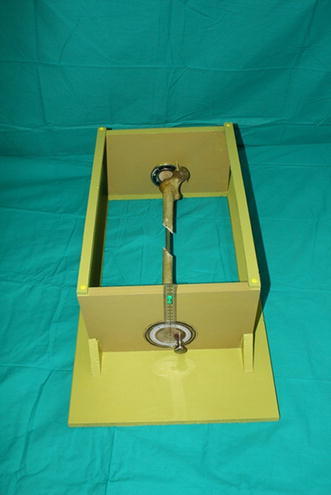
Pediatric femoral fracture model mounted in wooden frame

We developed a mathematical method to calculate fracture rotation from a direct roentgenogram. We obtained a well-preserved human cadaver femur and reamed its medulla starting from the tip of the greater trochanter down to the intercondylar notch. We performed an oblique midshaft osteotomy to create an oblique femoral fracture model. Then we inserted a wooden rod into the medullary cavity through the length of the proximal and distal fragments to function as the axis of rotation. We distracted the fracture ends to see the rotation more clearly. Then a wooden frame was constructed, and the bone and wooden rod were secured to the frame with metal pins. This system allowed the distal part of the fracture to rotate around the wooden rod while the proximal part was stable. The degree of rotation was measured with a goniometer, which was adapted to the frame (Fig. 1).

Digitized anteroposterior radiographs of the femur were obtained. Radiographs were taken with the fracture rotation ranging from the neutral position to 60° external rotation and to 60° internal rotation in 5° increments of rotation.

Three orthopedic surgeons and two orthopedic residents independently measured the radiographs. The observers were blinded to the protocol used to take the radiographs. They were allowed to use the photo management program of their choice and could modify the images to their liking.

**Fig. 2:**
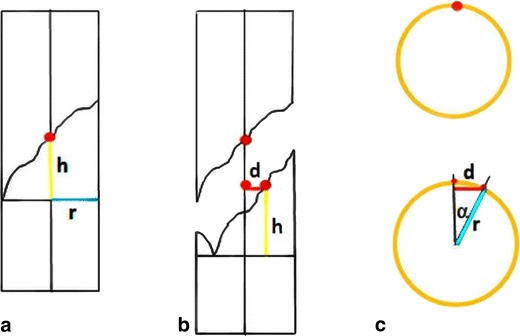
Schematic drawing of bone in frame. **a**, **b** Frontal view, **c** transverse view

**Fig. 3:**
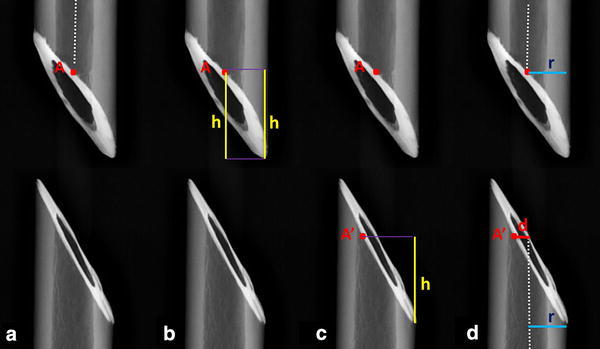
Step by step measurement technique. **a** The intersection of the proximal fracture line and the vertical midline of the femoral diaphysis is found (point *A*). **b** The vertical distance of this point to the fracture edge is determined (*h*). **c** The distance *h* is transferred to the distal fracture line edge; a line perpendicular to *h* is drawn. The intersection of this line with the distal fracture line is the point *A*’ (the corresponding point of *A*). **d** The distance between *A* and *A*’ is the distance *d*

**Fig. 4:**
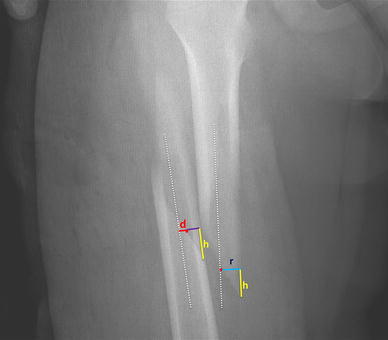
In this example fracture edges are clearly seen. Midline axes of proximal and distal fragments are drawn. The intersection point of proximal fracture line and proximal midline axis is determined. Vertical distance of this point to the fracture edge is *h*. This distance is transfered to the distal fragment. Note that each *h* line is parallel to the corresponding midline axis. The corresponding point on the distal fracture line is determined. Its horizontal distance to the distal midline axis is *d*. α = sin^−1^(*d*/*r*), α = sin^−1^(7/20), α = 20.5°

**Fig. 5:**
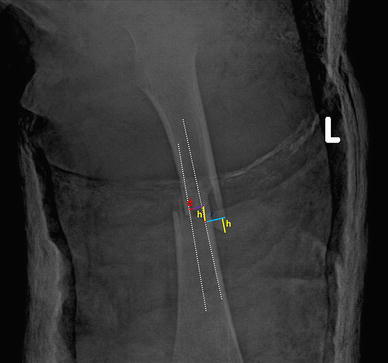
This example is more complicated because of casting and overlapping of fracture lines. According to the same principles, *lines* and *points* are drawn. Note that the intersection point of the proximal fracture line and proximal midline axis is overlapping with the distal fracture edge. α = sin^−1^(*d*/*r*), α = sin^−1^(6/32), α = 10.8°

Our method of calculating the rotation is based upon the displacement of a certain point on the distal fracture fragment on a horizontal line. For easier measurement, we chose this point as the intersection of the fracture line and the vertical midline of the femoral diaphysis (the axis of rotation) (Fig. 2a). When the distal fragment rotates, this point moves on a horizontal line for a distance of *d*, while its perpendicular distance *h* to the fracture edge does not change (Fig. 2b). On the transverse plane, the point rotates around the radius *r* for an angle of α (Fig. 2c). As the distance *d* and the radius *r* can be measured from the direct roentgenogram (Fig. 3), the angle α can be calculated from the equation sin α = *d*/*r*. The equation is rearranged as: α = sin^−1^(*d*/*r*). Figure 3 represents the step-by-step measurement technique. Examples of the measurement technique for two different fracture patterns are given in Figs. 4 and 5.

The angles of rotation were calculated with this equation by five observers. We used a calculator to perform a sin^−1^ function. Since a calculator may not be available everywhere, a list of ratios and corresponding angles was prepared (Table 1) to facilitate the use of this method in the clinical setting.

**Table 1:** Ratios and corresponding rotation angles

*r*/*d*	Rotation angle (°)
11.47	5
5.75	10
3.86	15
2.92	20
2.36	25
2	30
1.74	35
1.55	40
1.41	45
1.30	50
1.22	55
1.15	60

In order to deal with larger numbers, ratios are given as *r*/*d*

All statistical calculations were performed using SPSS for Windows, version 20.0. Accuracy analysis was performed by calculating absolute and relative errors. Pearson’s correlation coefficient was used to determine the correlation between the calculated rotation and the actual rotation. Interobserver reliability was quantified by calculating the intraclass correlation coefficient.

## Results

The mean calculated rotation values for five observers are given in Table 2. Calculated rotation values were close to actual rotation values throughout the arc of rotation from 60° of external rotation to 60° of internal rotation. The mean absolute error of five observers for all measurements of external and internal rotation was 3.97° (±0.83). From 25° of external rotation to 45° of internal rotation, the calculated rotation values were within 5° of the actual rotation. Mean absolute error increased as the amount of rotation increased. This increase was not seen in mean relative error.

**Table 2:** Calculated values

Actual rotation (AR) (°)	Mean calculated rotation (MCR) (°)		Mean absolute error (MAE) (°)		Mean relative error (MRE)	
	ER	IR	ER	IR	ER	IR
5	4.88	6.08	1.16	1.08	0.23	0.21
10	9.81	10.45	0.88	0.98	0.08	0.09
15	17.31	14.40	2.31	1.44	0.15	0.09
20	23.95	19.49	3.95	1.35	0.18	0.06
25	29.18	26.61	4.18	2.03	0.16	0.08
30	35.86	30.21	5.86	1.62	0.19	0.05
35	41.45	34.17	6.45	1.38	0.18	0.03
40	45.97	37.86	5.97	4.14	0.14	0.10
45	51.06	41.99	7.57	3.01	0.16	0.06
50	57.57	46.10	7.57	5.55	0.15	0.11
55	63.17	47.83	8.17	7.17	0.14	0.13
60	67.36	52.32	7.36	7.68	0.12	0.12

MAE = ; MRE =

*ER* external rotation, *IR* internal rotation

**Fig. 6:**
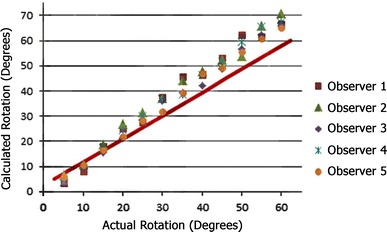
Calculated rotation versus actual rotation (external rotation)

**Fig. 7:**
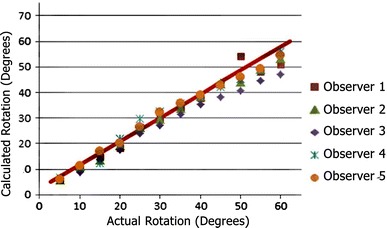
Calculated rotation versus actual rotation (internal rotation)

The accuracy of calculated rotation varied uniformly for all observers for both external and internal rotation measurements (Figs. 6, 7). The correlation coefficient between calculated and actual rotation values was 0.9927. Correlation was significant at the 0.01 level. The interobserver intraclass correlation coefficient for calculated rotation was 0.997.

## Discussion

Rotational deformity is a problem in children with femoral shaft fractures who are treated with closed reduction and spica casting [14, 15]. It can be determined clinically by inspection of the limb alignment, with evaluation of hip range of motion and observation of the gait of the child. In the study by Saseendar et al. [13], 43.75 % of children treated with spica casting had in-toe gait. In order to prevent this complication, malrotation has to be determined early in the course of treatment. Measuring the antetorsion angle by computed tomography and comparing it with the opposite side has been used to assess malrotation during follow-up [7, 8]. Bulut et al. [6] used computed tomography to follow children treated with spica casting. They corrected the fractures with a rotational deformity greater than 10° with a gypsotomy through the level of fracture. Close monitoring of fracture reduction is important to be able to make an early intervention such as gypsotomy or surgery in the case of malrotation. Using computed tomography for follow-up exposes the child to much radiation and is expensive. With our measurement technique, a direct roentgenogram could become the technique of choice in imaging studies for follow-up of rotational alignment of pediatric femoral shaft fractures.

In this study we chose to perform the measurement technique on a femoral fracture model because the femur is a long tubular bone with a long cylindrical segment. Theoretically, our formula works if the fracture line is on a cylindrical bone segment. In other words, the transverse section of the bone should be round shaped (Fig. 2c). So, theoretically, our method can be performed on cylindrical portions of every long tubular bone. We focused on pediatric femoral fractures because they are treated conservatively more commonly than adult femoral fractures.

But malrotation may also be a problem for surgically stabilized pediatric or adult femoral fractures [18]. Rotational control is difficult, especially in semi-closed approaches such as intramedullary (IM) nailing and minimally invasive percutaneous osteosynthesis (MIPO), where anatomical reduction under direct vision is impossible [19]. Incidences of rotational malalignment have been reported between 20 and 30 % in IM nailing and up to 38.5 % in the MIPO technique [19–21]. Several intraoperative methods have been described to overcome this problem. Langer et al. [22] used the cortical step sign as a tool for assessing rotational deformity during IM nailing. Braten et al. [23] measured the angle between the horizontal plane and the central head–neck axis and used this angle as a guide to intraoperative rotational reduction. Jaarsma et al. [19] used the lesser trochanter as a landmark and tried to obtain the mirror view of the contralateral side to avoid rotational malalignment. Ehrenstein et al. [24] defined an ultrasound-based method that could be used intraoperatively during IM nailing of femoral fractures. Fluoroscopic techniques are difficult to use because exact positioning of the patient is necessary. We believe that our method can also measure the rotation in the presence of an implant if the implant does not overlap the fracture site. It can also be used intraoperatively to assess rotational alignment, especially external fixation, the MIPO technique and elastic IM nailing.

Measuring the rotation of a fracture within 5° of the actual rotation has been accepted as successful [17]. Our mean absolute error was 3.97° for all measurements. There was good correlation between actual rotation and calculated rotation values of all observers, and the intraclass correlation coefficient was 0.997. These findings indicate that this method is accurate and reliable. The accuracy is higher at lower degrees of rotation, and it decreases as the amount of rotation increases. This is probably due to the decreased amount of change on parameter *d* at higher amounts of rotation.

Rotational deformity exceeding 10° is found to be symptomatic and is considered to be unacceptable in many studies [6, 13–15]. Malrotation of more than 30° is much more symptomatic, and this amount of rotation can easily be recognized with inspection. So it is critical to diagnose a malrotation between 10° and 30°. The accuracy of our method in the first 30° of rotation is better than the overall accuracy. Therefore, it can be used safely for the follow-up of pediatric femoral shaft fractures. If there is a malrotation exceeding 10°, an early intervention can be performed.

In our model, the fracture is only rotated and not translated or angulated. But angulation on the anteroposterior plane and translation do not change our measurement, because the parameters in the formula (*h*, *d* and *r*) do not change. On the other hand, if a fracture is angulated on the lateral plane, the parameter *h* changes, and its new value can be determined on the lateral radiograph according to the amount of angulation, or the measurement can be performed directly on the lateral radiograph if there is no angulation on the anteroposterior (AP) radiograph. So in the application of this method in the clinical setting, AP and lateral radiographs should be taken. If there is no angulation on the lateral plane, measurement can safely be made on the AP radiograph and vice versa.

The fracture in our model is an oblique osteotomy, and the fracture ends are smooth. In a real fracture, such smoothness does not exist, and there are spikes on the fracture ends. These spikes can be used as landmarks in rotation measurements. In this case, measurement of parameter *h* is not required. This is particularly important in transverse fractures because, in these fractures, parameter *h* is very small (near zero), and measurement is difficult. With our landmark technique, transverse fracture rotations can be measured more easily.

Fracture comminution may preclude accurate measurement with this method if the comminution is severe. But if there is only a butterfly fragment or there is a segmental fracture, we can still calculate the overall rotation by measuring the rotation of the fragments with reference to each other.

A good radiologic technique is important for the highest accuracy using our method. We prefer using digitized roentgenograms and magnification programs. If digitized roentgenograms are not available, scans or digitized photographs of standard roentgenograms can be taken and magnification can be performed on a computer. Magnification quality of the roentgenogram is also very important. Resolution should be sufficient to allow adequate magnification. The more magnification that can be performed, the more precise is the measurement. Fracture ends have to be clearly visible.

To the best of our knowledge, this is the first study in the literature that defines a method of calculating fracture malrotation of a long tubular bone from a simple direct roentgenogram. Techniques for quantifying supracondylar humerus fracture malrotation have been described previously [17, 25]. Our method is defined on a femoral fracture model, but, theoretically, it can also be performed on other long tubular fractures. Further clinical studies that compare this method with computed tomography measurements would allow reaching firmer conclusions regarding the feasibility of this approach.

## References

[1] Wright JG (2000). The treatment of femoral shaft fractures in children: a systematic overview and critical appraisal of the literature. Can J Surg.

[2] Khazzam M, Tassone C, Liu XC, Lyon R, Freeto B, Schwab J, Thometz J (2009). Use of flexible intramedullary nail fixation in treating femur fractures in children. Am J Orthop.

[3] Flynn JM, Luedtke LM, Ganley TJ, Dawson J, Davidson RS, Dormans JP, Ecker ML, Gregg JR, Horn BD, Drummond DS (2004). Comparison of titanium elastic nails with traction and a spica cast to treat femoral fractures in children. J Bone Joint Surg Am.

[4] Flynn JM, Hresko T, Reynolds RA, Blasier RD, Davidson R, Kasser J (2001). Titanium elastic nails for pediatric femur fractures: a multicenter study of early results with analysis of complications. J Pediatr Orthop.

[5] Herring JA (2007). Tachdjian’s Pediatric Orthopaedics.

[6] Bulut S, Bulut O, Tas F, Egilmez H (2003). The measurement of the rotational deformities with computed tomography in femoral shaft fractures of the children treated with early spica cast. Eur J Radiol.

[7] Hermann KL, Egund N (1997). CT measurement of anteversion in the femoral neck. The influence of femur positioning. Acta Radiol.

[8] Staheli LT, Corbett M, Wyss C, King H (1985). Lower-extremity rotational problems in children. Normal values to guide management. J Bone Joint Surg Am.

[9] Dunlop K, Shands AR, Hollister LC, Gaul JS, Streit HA (1953). A new method for determination of torsion of the femur. J Bone Joint Surg Am.

[10] Henriksson L (1980). Measurement of femoral neck anteversion and inclination. Acta Orthop Scand (suppl).

[11] Norbeck DE, Asselmeier M, Pinzor MS (1990). Torsional malunion of a femur fracture: diagnosis and treatment. Orthop Rev.

[12] Davids JR (1994). Rotational deformity and remodeling after fracture of the femur in children. Clin Orthop.

[13] Saseendar S, Menon J, Patro DK (2010). Treatment of femoral fractures in children: is titanium elastic nailing an improvement over hip spica casting?. J Child Orthop.

[14] Staheli LT, Sheridan GW (1977). Early spica cast management of femoral shaft fractures in young children. Clin Orthop.

[15] Verbeek H (1979). Does rotational deformity following femur shaft fracture correct during growth?. Reconstr Surg Travmatol.

[16] Mansour AA, Wilmoth JC, Mansour AS, Lovejoy SA, Mencio GA, Martus JE (2010). Immediate spica casting of pediatric femoral fractures in the operating room versus the emergency department. Comparison of reduction, complications and hospital charges. J Pediatr Orthop.

[17] Henderson ER, Egol KA, Van Bosse HJP, Schweitzer ME, Pettrone SK, Feldman DS (2007). Calculation of rotational deformity in pediatric supracondylar humerus fractures. Skeletal Radiol.

[18] Heybeli M, Muratli HH, Celebi L, Gulcek S, Bicimoglu A (2004). The results of intramedullary fixation with titanium elastic nails in children with femoral fractures. Acta Orthop Traumatol Turc.

[19] Jaarsma RL, Verdonschot N, Van der Venne R, Van Kampen A (2005). Avoiding rotational malalignment after fractures of the femur by using the profile of the lesser trochanter: an in vitro study. Arch Orthop Trauma Surg.

[20] Jaarsma RL, Pakvis DFM, Verdonschot N, Biert J, Van Kampen A (2004). Rotational malalignment after intramedullary nailing of femoral fractures. J Orthop Trauma.

[21] Buckley R, Mohanty K, Malish D (2011). Lower limb malrotation following MIPO technique of distal femoral and proximal tibial fractures. Injury.

[22] Langer JS, Gardner MJ, Ricci WM (2010). The cortical step sign as a tool for assessing and correcting rotational deformity in femoral shaft fractures. J Orthop Trauma.

[23] Braten M, Tveit K, Junk S, Aamodt A, Anda S, Terjesen T (2000). The role of fluoroscopy in avoiding rotational deformity of treated femoral shaft fractures: an anatomical and clinical study. Injury.

[24] Ehrenstein T, Rikli DA, Peine R, Gutberlet M, Mittlmeier T, Banzer D, Maurer J, Felix R (1999). A new ultrasound-based method for the assessment of torsional differences following closed intramedullary nailing of femoral fractures. Skel Radiol.

[25] Gordon JE, Patton CM, Luhmann SJ, Bassett GS, Schoenecker PL (2001). Fracture stability after pinning of displaced supracondylar distal humerus fractures in children. J Pediatr Orthop.

